# Compromised dynamic cerebral autoregulation in patients with generalized anxiety disorder: a study using transfer function analysis

**DOI:** 10.1186/s12888-018-1713-z

**Published:** 2018-06-01

**Authors:** Zhen-Ni Guo, Shan Lv, Jia Liu, Zan Wang, Hang Jin, Quanli Qiu, Xin Sun, Yi Yang

**Affiliations:** 1 Department of Neurology, The First Hospital of Jilin University, Xinmin Street 71#, Chang Chun, 130021 China; 2 Clinical Trail and Research Center for Stroke, Department of Neurology, The First Hospital of Jilin University, Chang Chun, China; 3 Shenzhen Institutes of Advanced Technology, Chinese Academy of Sciences, Xueyuan Avenue, Shenzhen University Town, Shenzhen, China

**Keywords:** Generalized anxiety disorder, Dynamic cerebral autoregulation, Transcranial Doppler

## Abstract

**Background:**

Patients with generalized anxiety disorder (GAD) usually present with various neurological symptoms, but the mechanisms remain unclear. We aimed to analyze the characteristics of dynamic cerebral autoregulation (dCA) in patients with GAD.

**Methods:**

Patients (aged ≥18 years) who were diagnosed with GAD were enrolled in this study. Medically and psychiatrically healthy volunteers were recruited as controls. Subjects received the Hamilton Rating Scale for Anxiety (HAMA) and 17-item Hamilton Depression Rating Scale (HAMD) evaluation. Noninvasive continuous arterial blood pressure and bilateral middle cerebral artery blood flow velocity were recorded simultaneously from each subject. Transfer function analysis was used to derive the autoregulatory parameters, including phase difference, gain, and coherence function.

**Results:**

A total of 57 patients with GAD and 40 healthy volunteers were enrolled. We found that the phase difference values were significantly compromised in patients with GAD. In the Spearman correlation analysis, the phase difference values were negatively correlated with the HAMA scores and the HAMD scores. In the multiple linear regression analysis, GAD is negatively correlated with the phase difference values, whereas age is positively correlated with the phase difference values.

**Conclusions:**

Our results suggested that the dCA was compromised in patients with GAD and negatively correlated with the score of anxiety. Improving the dCA may be a potential therapeutic method for treating the neurological symptoms of GAD patients.

## Background

Generalized anxiety disorder (GAD) is one of the most common mental disorders in the world [1, 2] and can negatively affect the life quality of patients and disrupt important activities of daily living [1]. Patients with GAD usually present with various neurological symptoms, such as dizziness, headache, and sleep disorders; the mechanisms of these symptoms, however, remain unclear. It has been reported that in patients with GAD, the cerebral hemodynamics show abnormal manifestations [3, 4], which may be a reason for the neurological symptoms of GAD.

**Fig. 1:**
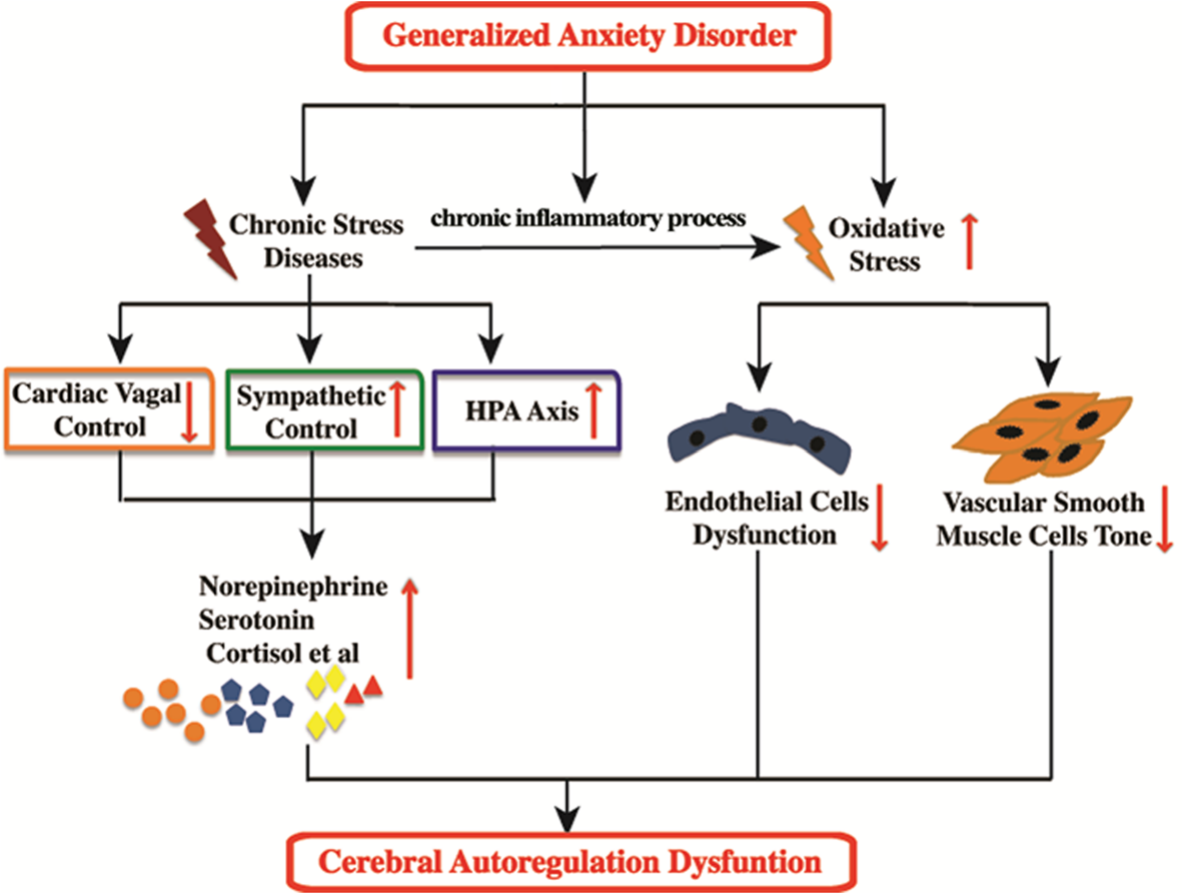
Hypothesis between generalized anxiety disorder (GAD) and cerebral autoregulation impairment. In patients with GAD, neuroregulation, myogenic response, and endothelial regulation may be dysfunctional, which could damage cerebral autoregulation

Cerebral autoregulation, which protects the brain tissue from hyperperfusion or hypoperfusion, is critical in regulating cerebral hemodynamics and has been found to play an important role in many neurological diseases [5–7]. Previous studies found that the factors involved in cerebral autoregulation regulation, such as neuroregulation, myogenic response, and endothelial regulation, etc., are dysfunctional in patients with GAD (Fig. 1) [8–12]. Our previous study also showed that patients with GAD cannot maintain normal cerebral blood flow velocity from supine to standing [13]. These data imply that cerebral autoregulation may be impaired in GAD patients.

Cerebral autoregulation is divided into static cerebral autoregulation and dynamic cerebral autoregulation (dCA), and the dCA is more sensitive to pathological situations [14, 15]. When dCA is measured, continuous cerebral blood flow velocities (assessed using transcranial Doppler) and continuous finger blood pressure (assessed using a servo-controlled plethysmograph) were recorded simultaneously. These data were then analyzed using transfer function analysis, an approach used to analyze dCA based on spontaneous fluctuations of blood pressure and cerebral blood flow velocities at rest [16, 17]. In previous studies, dCA calculated by transfer function analysis has been applied in healthy subjects [18], cerebrovascular disease [5, 19], cognitive impairment [6], etc. However, dCA has not been studied in patients with GAD.

In this study, we hypothesize that dCA is compromised in patients with GAD. If our hypothesis is valid, dCA may serve as a potential therapeutic target to improve the neurological symptoms in patients with GAD.

## Methods

### Participants

The prospective study design was approved by the ethics committee of the First Hospital of Jilin University under the guidelines of the Helsinki Declaration of 1975/1983. Written informed consent was obtained from all subjects. Patients whose chief complaint was poor sleep were selected for screening. Patients (aged ≥18 years) who met the *Diagnostic and Statistical Manual of Mental Disorders, Fourth Edition, Text Revision* criteria for GAD [20] were recruited from May 2016 to November 2016 in the outpatient unit of the Neurological Department. Patients who met Diagnostic and Statistical Manual of Mental Disorders criteria for Major Depressive Disorder were excluded. Patients were otherwise healthy, with no ascertained disorders in the nervous, cardiovascular, or respiratory systems and without hypertension, diabetes, or hyperlipidemia. The clinical workup consisted of laboratory tests (liver and kidney function tests, hematology profile, blood glucose tests, and blood lipid tests), blood pressure, electrocardiography, transcranial Doppler (EMS-9 PB, Delica, China), carotid ultrasound (IU22, Phillips, Andover, MA, USA), cranial computed tomography/magnetic resonance imaging, and physical examination. Patients were evaluated with the Hamilton Rating Scale for Anxiety (HAMA) [21] and the 17-item Hamilton Depression Rating Scale (HAMD) [22]. Medically and psychiatrically healthy volunteers were recruited as controls. Two blinded clinical psychiatrists evaluated the patients’ mental health status.

### Dynamic cerebral autoregulation (dCA) protocol

The examination of dCA was performed as reported in previous research [5, 7, 23]. Subjects were asked to avoid nicotine, caffeine, alcohol, and all kinds of sleep medicines for at least 24 h before the dCA examination. The examination was performed in a quiet, dedicated research room with minimal surrounding stimuli. First, the baseline arterial blood pressure was measured at the brachial artery using an automatic blood pressure monitor (Omron 711). Second, we simultaneously recorded continuous spontaneous arterial blood pressure on the middle finger using a servo-controlled plethysmograph (Finometer Pro, Netherlands) and continuous bilateral middle cerebral artery blood flow velocity at a depth of 45 mm to 60 mm with 2 MHz probes attached to a customized head frame (MultiDop X2, DWL, Sipplingen, Germany). End-tidal carbon dioxide was recorded using a capnograph with a facemask attached to the nasal cannula. Data were recorded for 10 min for further dCA examination analysis.

### Data analysis

The dCA analysis was performed as previously reported [5, 7] and was analyzed blindly for each subject. Briefly, dCA data were analyzed using MATLAB (MathWorks, Natick, MA, USA). Beat-to-beat alignment of the data was achieved with a cross-correlation function to eliminate possible time lags. The relationship between dynamic changes in spontaneous arterial blood pressure and bilateral middle cerebral artery blood flow velocity was assessed with a transfer function analysis. For each recording, arterial blood pressure and bilateral cerebral artery blood flow velocity were divided into a number of data segments by a 60-s window with a 30-s overlap. For one segment of arterial blood pressure and bilateral cerebral artery blood flow velocity, the transfer function analysis was implemented as,

1 $$ H(f)=\frac{S_{pv}(f)}{S_{pp}(f)}, $$

where *H*(*f*) denotes the frequency response. *S*_*pp*_(*f*) is the auto-spectrum of arterial blood pressure, and *S*_*pv*_(*f*) is the cross-spectrum between arterial blood pressure and cerebral artery blood flow velocity. For each subject, *S*_*pp*_(*f*) and *S*_*pv*_(*f*) were averaged over the segments to improve statistical reliability. The gain |*H*(*f*)| and phase difference *ϕ*(*f*) can then be computed as,

2 $$ \left|H(f)\right|=\sqrt{\left\{{\left|{H}_R(f)\right|}^2+{\left|{H}_I(f)\right|}^2\right\}}, $$

3 $$ \varnothing (f)={\tan}^{-1}\left[\frac{H_I(f)}{H_R(f)}\right], $$

where *H*_*R*_(*f*) and *H*_*I*_(*f*) are the real and imaginary parts of *H*(*f*), respectively. Low phase difference and high gain values at a low frequency band (0.06-0.12 Hz) indicate that cerebral artery blood flow velocity follows changes of arterial blood pressure passively, thus suggesting impairment of autoregulation [16, 17]. We also calculated coherence function to quantify the linearity in the frequency domain using a routine provided by Signal Processing Toolbox in MATLAB with the Welch method for the estimation of power spectral density and hamming window for the reduction of spectral leakage. The recordings with averaged coherence < 0.4 at the low frequency band were considered with insufficient linearity and therefore excluded from the transfer function analysis.

### Statistical analysis

The Statistical Package for the Social Sciences Version 17.0 (SPSS, IBM, West Grove, PA, USA) was used to analyze the data. Continuous data are expressed as mean and standard deviation. Comparison between two groups were analyzed using Student’s t-tests. The discrete variables are expressed as the rate (percentage) and were analyzed using chi-squared and Fisher’s exact tests. The Spearman correlation analysis was used to analyze the relationship between phase difference values and HAMA scores and the relationship between phase difference values and HAMD scores. Multiple linear regression analysis was used to explore the effects of covariates on phase or gain. Calculated two-tailed *P* values < 0.05 were considered statistically significant.

## Results

### Demographic information

In total, 57 patients with GAD (45.05 ± 14.83; 18 males) and 40 healthy volunteers were enrolled in the study. The baseline characteristics are presented in Table 1.

**Table 1:** Baseline characteristics, phase difference, and gain in the patients and controls

	GAD (*n* = 57)	Control (*n* = 40)	t /χ^2^	*p*
Male, n (%)	18 (31.58%)	24 (60.00%)	6.773	0.009
Age (years)	45.05 ± 14.83	43.10 ± 11.51	0.698	0.487
HAMA	19.79 ± 5.93	3.60 ± 1.71	19.501	< 0.001
HAMD	13.96 ± 4.08	4.37 ± 1.33	16.534	< 0.001
Mean ABP, mmHg	89.12 ± 7.48	86.60 ± 9.73	1.443	0.152
Heart rate	76.07 ± 8.99	74.10 ± 8.20	1.101	0.274
End-title CO_2_, mmHg	35.23 ± 3.12	35.16 ± 2.56	0.613	0.542
Phase difference, degree				
Left hemisphere	43.43 ± 14.39	55.00 ± 8.86	−4.891	< 0.001
Right hemisphere	43.30 ± 15.40	54.07 ± 9.36	− 4.273	< 0.001
Gain, %/%				
Left hemisphere	0.89 ± 0.31	0.85 ± 0.24	0.820	0.414
Right hemisphere	0.86 ± 0.29	0.86 ± 0.22	−0.064	0.949
Smoking, n (%)	12 (21.1)	7 (17.5)	0.188	0.664
Drinking, n (%)	4 (7.0)	1 (2.5)	0.646	0.310

*GAD* generalized anxiety disorder, *ABP* arterial blood pressure, *HAMA* Hamilton Rating Scale for Anxiety, *HAMD* Hamilton Depression Rating Scale

### Dynamic cerebral autoregulation

#### GAD patients

**Fig. 2:**
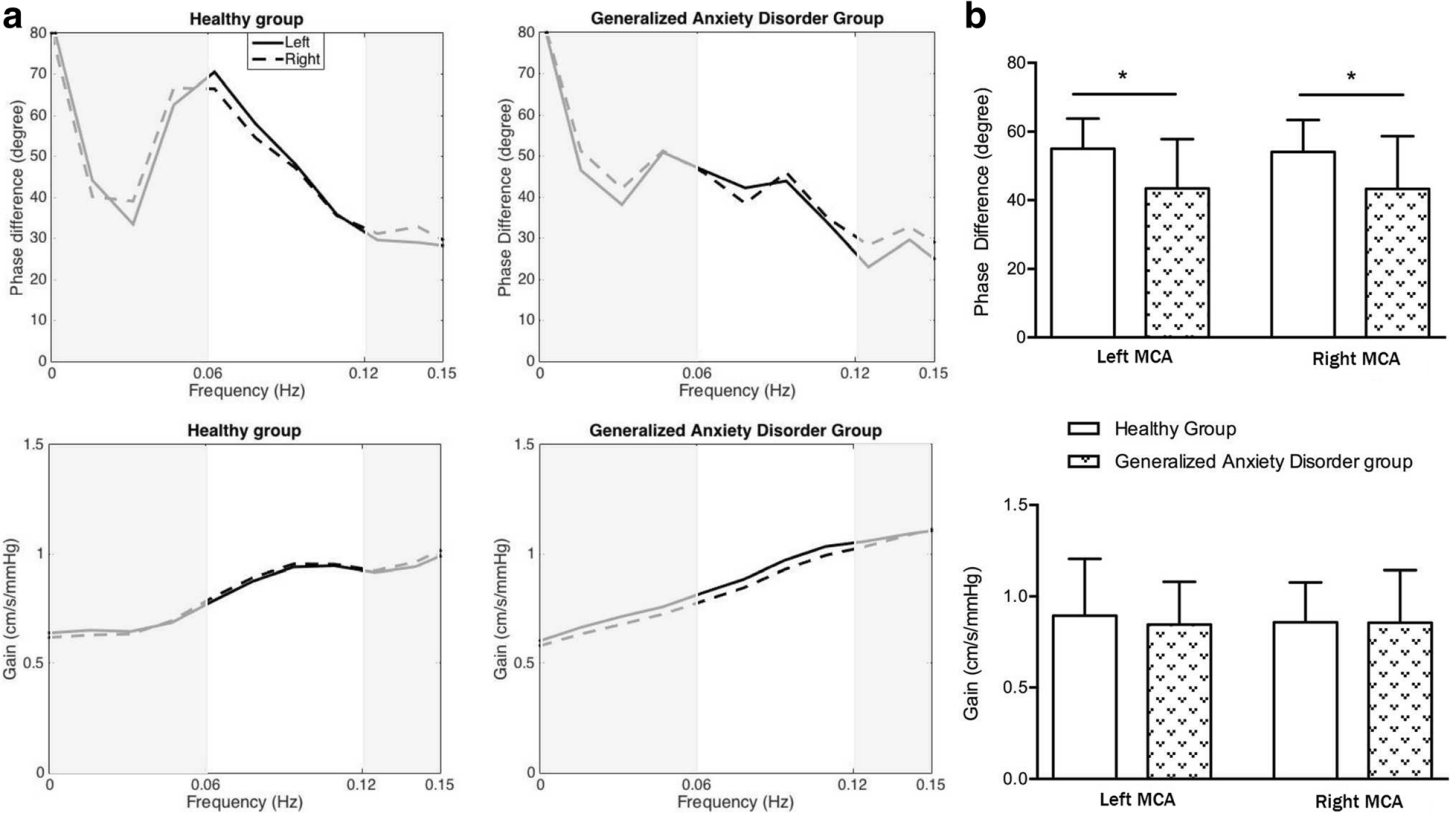
**a** The phase difference and gain derived from the transfer function within significant interval 0.06-0.12 Hz are plotted. (−: left middle cerebral artery [MCA] and ---: right MCA). Phase difference values (parameter of dynamic cerebral autoregulation) were significant compromised in patients with generalized anxiety disorder (GAD) compared with the healthy group, indicating an impairment of dynamic cerebral autoregulation in patients with GAD. There was no difference of the gain values between GAD group and healthy group. **b** Statistical analysis of phase differences and gains are shown. Phase difference values in the GAD group were significantly lower than the corresponding MCA of the healthy group. There was no difference in gain values in the two groups

The patients with GAD showed no difference in phase difference values between the left and right hemispheres. However, when compared with the healthy controls, the phase difference values of both hemispheres of GAD patients were significantly lower than the corresponding hemisphere of the healthy controls. In addition, there was no significant difference in the gain values between GAD patients and healthy controls in both the left and right hemispheres (Fig. 2, and Table 1).

#### Correlation analysis

In the Spearman correlation analysis, both the left and right phase difference values were negatively correlated to HAMA scores (left: *r* = − 0.365, *p* < 0.001; right: *r* = − 0.348, *p* < 0.001). Similarly, both the left and right phase difference values were negatively correlated to HAMD scores (left: *r* = − 0.350, *p* < 0.001; right: *r* = − 0.363, *p* < 0.001).

#### Multiple linear regression analysis

The associations between clinical factors and phase difference are shown in Table 2. GAD and age are found to be correlated with phase difference values. GAD is negatively correlated to phase difference, whereas age is positively correlated to phase difference after adjusting for covariates (Table 2). No factors were detected associated with gain.

**Table 2:** Multiple regression Coefficients for mean phase difference values of left and right hemisphere

Covariates	Unstandardized coefficients		Standardized coefficients	95% CI for β		*P*
	β	Std. Error		Lower Bound	Upper Bound	
Constant	63.819	21.494		21.105	106.534	0.004
Age (years)	0.240	0.092	0.248	0.057	0.422	0.011
Mean ABP, mmHg	−0.063	0.150	−0.041	−0.362	0.236	0.676
Heart rate	−0.075	0.143	−0.050	−0.360	0.210	0.604
End-title CO_2_, mmHg	−0.229	0.389	−0.057	−1.003	0.544	0.557
Groups of mental disorder						
Control	Reference					
Generalized anxiety disorder	−11.052	2.629	−0.418	− 16.277	− 5.840	< 0.001
Gender						
Female	Reference					
Male	−0.143	3.024	0.005	−6.154	5.828	0.962
Smoking						
No	Reference					
Yes	−2.561	3.672	−0.078	−9.858	4.737	0.487
Drinking						
No	Reference					
Yes	−2.943	5.854	−0.050	−14.576	8.690	0.616

## Discussion

In the present study, we found that the dCA of both hemispheres in patients with GAD was significantly lower as compared with the healthy controls. In addition, the dCA function is negatively correlated with the anxiety score. Impaired dCA may be a mechanism underlying the neurological symptoms of GAD and thus may serve as a potential therapeutic target to alleviate the neurological symptoms in patients with GAD.

Generally, phase difference between arterial blood pressure and cerebral artery blood flow velocity at a certain frequency can be considered as time delay between these recordings. Therefore, lower phase difference (shorter time delay) indicates that blood flow changes in pace (passively) with fluctuations of blood pressure, suggesting that the distal arterioles and capillary do not respond to the changes of blood pressure. In contrast, notable phase difference (larger time delay) suggests that the phase of arterial blood pressure is outpaced by the phase of cerebral artery blood flow velocity, implying that the distal arterioles and capillary do not comply with the changes in blood pressure. The reason why patients with GAD present with impaired dCA remains unclear and needs further investigation. As a chronic stress disease, lower cardiac vagal control and hyperactivity of the sympathetic and hypothalamic-pituitary-adrenal axis were reported in patients with GAD [24–29], leading to disorganized secretion of norepinephrine, serotonin, cortisol, etc. Some of these neuroendocrine substances are vasoactive substances that regulate cerebral autoregulation (Fig. 1).

The integrity of structure and function of the endothelium is essential to maintain a functional cerebral autoregulation [30–33]. However, in patients with GAD, both the structure and function of the endothelium may be damaged due to oxidative stress, which is another characteristic of GAD [34–36]. In addition, CRP [37], TNF-α, and IL-17 [38, 39] are increased in patients with GAD, indicating that the inflammatory process is activated and can induce endothelial cell dysfunction. Furthermore, oxidative stress can alter the vascular smooth muscle tone, another indispensable mechanism in regulating cerebral autoregulation, by changing reactive oxide species concentration (Fig. 1) [40].

As described above, the changes in neuroregulation, endothelial regulation, and myogenic response may collectively result in the impairment of dCA, leading to unstable cerebral blood flow in patients with GAD. A previous study by Kalk, et al. supports our findings [3]. They found that patients with untreated GAD showed increased perfusion in the left Broca’s area and left occipitotemporal region, and venlafaxine-treated GAD patients showed increased cerebellar perfusion bilaterally [3]. In the present study, the impairment of dCA can result in abnormal cerebral perfusion. Our previous study also yielded some meaningful results: we found that patients with anxiety showed more pronounced decreases in cerebral blood flow velocity with abrupt standing, which indicates impaired dCA [4].

It is worth mentioning that phase difference values were negatively correlated with the HAMA scores, which suggests that as the HAMA scores increase, the phase difference values, i.e., dCA, tend to decrease. In addition, the negative correlation between phase difference values and HAMD scale suggests a potential impact of depressive symptoms on phase difference values. This phenomenon deserves further study in patients with major depressive disorder. The impairment of dCA in patients with GAD indicates that cerebral vascular function is a therapeutic target of GAD. Thus, methods to improve dCA may potentially relieve the neurological symptoms in patients with GAD.

Both the studies from Ortega-Gutierrez and Yams suggested that dCA remains intact in the elderly, though their intracranial arteries may be affected by atherosclerosis [41, 42]. In our study, we found age is a weak positive correlation to phase difference; the causes are not clear. One possible reason is that the patients we included were relatively young, and the age span is relatively small. However, this explanation is inadequate.

This study has some limitations. The first is the gender mismatch of the GAD group and controls. Because gender affect cerebral blood flow via complex mechanisms [43], we could not rule out the possible influence of sex on dCA. However, it is worth mentioning that the regression analyses suggest no effect of gender in our study. Second, we do not have the neuroendocrine results of our patients’ blood to further support our results. Third, this is an observational study without in-depth mechanism research. Furthermore, large sample sizes and animal studies are needed. In addition, medication condition was not included in this article, which could potentially influence the results.

## Conclusions

Our results suggested that the dCA was compromised in patients with GAD and negatively correlated with the score of anxiety. Improving the dCA may be a potential therapeutic method for treating the neurological symptoms of GAD patients.

## Abbreviations

DCA: Dynamic cerebral autoregulation
GAD: Generalized anxiety disorder
HAMA: Hamilton rating scale for anxiety
HAMD: Hamilton Depression Rating Scale

## References

[1] Locke AB, Kirst N, Shultz CG (2015). Diagnosis and management of generalized anxiety disorder and panic disorder in adults. Am Fam Physician.

[2] Phillips MR, Zhang J, Shi Q, Song Z, Ding Z, Pang S, Li X, Zhang Y, Wang Z (2009). Prevalence, treatment, and associated disability of mental disorders in four provinces in China during 2001-05: an epidemiological survey. Lancet.

[3] Kalk NJ, Melichar J, Holmes RB, Taylor LG, Daglish MR, Hood S, Edwards T, Lennox-Smith A, Lingford-Hughes AR, Nutt DJ (2012). Central noradrenergic responsiveness to a clonidine challenge in generalized anxiety disorder: a single photon emission computed tomography study. J Psychopharmacol.

[4] Zhang HL, Guo ZN, Yang G, Yang L, Han K, Wu J, Xing Y, Yang Y (2012). Compromised cerebrovascular modulation in chronic anxiety: evidence from cerebral blood flow velocity measured by transcranial Doppler sonography. Neurosci Bull.

[5] Ma H, Guo ZN, Liu J, Xing Y, Zhao R, Yang Y (2016). Temporal course of dynamic cerebral autoregulation in patients with intracerebral hemorrhage. Stroke.

[6] Tarumi T, Dunsky DI, Khan MA, Liu J, Hill C, Armstrong K, Martin-Cook K, Cullum CM, Zhang R (2014). Dynamic cerebral autoregulation and tissue oxygenation in amnestic mild cognitive impairment. J Alzheimer's Dis : JAD.

[7] Guo ZN, Xing Y, Wang S, Ma H, Liu J, Yang Y (2015). Characteristics of dynamic cerebral autoregulation in cerebral small vessel disease: diffuse and sustained. Sci Rep.

[8] Paulson OB, Strandgaard S, Edvinsson L (1990). Cerebral autoregulation. Cerebrovasc Brain Metab Rev.

[9] Makovac E, Meeten F, Watson DR, Herman A, Garfinkel SN, Critchley HD, Ottaviani C (2016). Alterations in amygdala-prefrontal functional connectivity account for excessive worry and autonomic dysregulation in generalized anxiety disorder. Biol Psychiatry.

[10] Fisher AJ, Newman MG (2013). Heart rate and autonomic response to stress after experimental induction of worry versus relaxation in healthy, high-worry, and generalized anxiety disorder individuals. Biol Psychol.

[11] Osika W, Montgomery SM, Dangardt F, Wahrborg P, Gan LM, Tideman E, Friberg P (2011). Anger, depression and anxiety associated with endothelial function in childhood and adolescence. Arch Dis Child.

[12] Munk PS, Isaksen K, Bronnick K, Kurz MW, Butt N, Larsen AI (2012). Symptoms of anxiety and depression after percutaneous coronary intervention are associated with decreased heart rate variability, impaired endothelial function and increased inflammation. Int J Cardiol.

[13] Guo ZN, Feng L, Yan X, Yang L, Huang S, Xing Y, Yang Y (2017). Characteristics of cardio-cerebrovascular modulation in patients with generalized anxiety disorder: an observational study. BMC Psychiatry.

[14] Dawson SL, Blake MJ, Panerai RB, Potter JF (2000). Dynamic but not static cerebral autoregulation is impaired in acute ischaemic stroke. Cerebrovasc Dis.

[15] Dawson SL, Panerai RB, Potter JF (2003). Serial changes in static and dynamic cerebral autoregulation after acute ischaemic stroke. Cerebrovasc Dis.

[16] Zhang R, Zuckerman JH, Giller CA, Levine BD (1998). Transfer function analysis of dynamic cerebral autoregulation in humans. Am J Phys.

[17] Reinhard M, Roth M, Muller T, Czosnyka M, Timmer J, Hetzel A (2003). Cerebral autoregulation in carotid artery occlusive disease assessed from spontaneous blood pressure fluctuations by the correlation coefficient index. Stroke.

[18] Barnes SC, Ball N, Haunton VJ, Robinson TG, Panerai RB (2017). The cerebrocardiovascular response to periodic squat-stand maneuvers in healthy subjects: a time-domain analysis. Am J Physiol Heart Circ Physiol.

[19] Chi NF, Ku HL, Wang CY, Liu Y, Chan L, Lin YC, Peng CK, Novak V, Hu HH, Hu CJ (2017). Dynamic cerebral autoregulation assessment using extracranial internal carotid artery Doppler ultrasonography. Ultrasound Med Biol.

[20] American psychiatric association. Diagnostic and statistical manual of mental disorders, 4th., text revision. Washington, DC: American Psychiatric Association. 2000.

[21] Hamilton M (1959). The assessment of anxiety states by rating. Br J Med Psychol.

[22] Hamilton M (1960). A rating scale for depression. J Neurol Neurosurg Psychiatry.

[23] Guo ZN, Xing Y, Liu J, Wang S, Yan S, Jin H, Yang Y (2014). Compromised dynamic cerebral autoregulation in patients with a right-to-left shunt: a potential mechanism of migraine and cryptogenic stroke. PLoS One.

[24] Thayer JF, Friedman BH, Borkovec TD (1996). Autonomic characteristics of generalized anxiety disorder and worry. Biol Psychiatry.

[25] Cameron OG, Smith CB, Lee MA, Hollingsworth PJ, Hill EM, Curtis GC (1990). Adrenergic status in anxiety disorders: platelet alpha 2-adrenergic receptor binding, blood pressure, pulse, and plasma catecholamines in panic and generalized anxiety disorder patients and in normal subjects. Biol Psychiatry.

[26] Reeves JW, Fisher AJ, Newman MG, Granger DA (2016). Sympathetic and hypothalamic-pituitary-adrenal asymmetry in generalized anxiety disorder. Psychophysiology.

[27] Dieleman GC, Huizink AC, Tulen JH, Utens EM, Creemers HE, van der Ende J, Verhulst FC (2015). Alterations in HPA-axis and autonomic nervous system functioning in childhood anxiety disorders point to a chronic stress hypothesis. Psychoneuroendocrinology.

[28] Lenze EJ, Mantella RC, Shi P, Goate AM, Nowotny P, Butters MA, Andreescu C, Thompson PA, Rollman BL (2011). Elevated cortisol in older adults with generalized anxiety disorder is reduced by treatment: a placebo-controlled evaluation of escitalopram. Am J Geriatr Psychiatry.

[29] Mantella RC, Butters MA, Amico JA, Mazumdar S, Rollman BL, Begley AE, Reynolds CF, Lenze EJ (2008). Salivary cortisol is associated with diagnosis and severity of late-life generalized anxiety disorder. Psychoneuroendocrinology.

[30] Ainslie PN, Murrell C, Peebles K, Swart M, Skinner MA, Williams MJ, Taylor RD (2007). Early morning impairment in cerebral autoregulation and cerebrovascular CO2 reactivity in healthy humans: relation to endothelial function. Exp Physiol.

[31] Guo ZN, Shao A, Tong LS, Sun W, Liu J, Yang Y (2016). The role of nitric oxide and sympathetic control in cerebral autoregulation in the setting of subarachnoid hemorrhage and traumatic brain injury. Mol Neurobiol.

[32] White RP, Vallance P, Markus HS (2000). Effect of inhibition of nitric oxide synthase on dynamic cerebral autoregulation in humans. Clin Sci.

[33] Preckel MP, Leftheriotis G, Ferber C, Degoute CS, Banssillon V, Saumet JL (1996). Effect of nitric oxide blockade on the lower limit of the cortical cerebral autoregulation in pentobarbital-anaesthetized rats. Int J Microcirc Clin Exp.

[34] Emhan A, Selek S, Bayazit H, Fatih Karababa I, Kati M, Aksoy N (2015). Evaluation of oxidative and antioxidative parameters in generalized anxiety disorder. Psychiatry Res.

[35] Kaya MC, Bez Y, Karababa IF, Emhan A, Aksoy N, Bulut M, Gunes M, Atli A, Selek S (2013). Decreased serum sulphydryl levels as a sign of increased oxidative stress in generalized anxiety disorder. Psychiatry Investig.

[36] Bulut M, Selek S, Bez Y, Karababa IF, Kaya MC, Gunes M, Emhan A, Aksoy N, Sir A (2013). Reduced PON1 enzymatic activity and increased lipid hydroperoxide levels that point out oxidative stress in generalized anxiety disorder. J Affect Disord.

[37] Copeland WE, Shanahan L, Worthman C, Angold A, Costello EJ (2012). Generalized anxiety and C-reactive protein levels: a prospective, longitudinal analysis. Psychol Med.

[38] Vieira MM, Ferreira TB, Pacheco PA, Barros PO, Almeida CR, Araujo-Lima CF, Silva-Filho RG, Hygino J, Andrade RM, Linhares UC (2010). Enhanced Th17 phenotype in individuals with generalized anxiety disorder. J Neuroimmunol.

[39] Michopoulos V, Powers A, Gillespie CF, Ressler KJ, Jovanovic T (2017). Inflammation in fear- and anxiety-based disorders: PTSD, GAD, and beyond. Neuropsychopharmacology.

[40] Faraci FM (2006). Reactive oxygen species: influence on cerebral vascular tone. J Appl Physiol.

[41] Yam AT, Lang EW, Lagopoulos J, Yip K, Griffith J, Mudaliar Y, Dorsch NW (2005). Cerebral autoregulation and ageing. J Clin Neurosci.

[42] Ortega-Gutierrez S, Petersen N, Masurkar A, Reccius A, Huang A, Li M, Choi JH, Marshall RS (2014). Reliability, asymmetry, and age influence on dynamic cerebral autoregulation measured by spontaneous fluctuations of blood pressure and cerebral blood flow velocities in healthy individuals. J Neuroimaging.

[43] Liu W, Lou X, Ma L (2016). Use of 3D pseudo-continuous arterial spin labeling to characterize sex and age differences in cerebral blood flow. Neuroradiology.

